# Optineurin restrains IL-17–associated neuroinflammation in trigeminal ganglia to preserve sensory function after ocular HSV-1 infection

**DOI:** 10.1093/jimmun/vkag161

**Published:** 2026-07-03

**Authors:** Joshua Ames, Rashmi Kadam, Tejabhiram Yadavalli, Chandrashekhar Patil, Ilina Bhattacharya, Deepak Shukla

**Affiliations:** Department of Ophthalmology and Visual Sciences, College of Medicine, University of Illinois Chicago, Chicago, IL, United States; Department of Microbiology and Immunology, College of Medicine, University of Illinois Chicago, Chicago, IL, United States; Department of Immunology, University of Washington, Seattle, WA, United States; Department of Ophthalmology and Visual Sciences, College of Medicine, University of Illinois Chicago, Chicago, IL, United States; Department of Ophthalmology and Visual Sciences, College of Medicine, University of Illinois Chicago, Chicago, IL, United States; Department of Ophthalmology and Visual Sciences, College of Medicine, University of Illinois Chicago, Chicago, IL, United States; Department of Ophthalmology and Visual Sciences, College of Medicine, University of Illinois Chicago, Chicago, IL, United States; Department of Microbiology and Immunology, College of Medicine, University of Illinois Chicago, Chicago, IL, United States; Department of Ophthalmology and Visual Sciences, College of Medicine, University of Illinois Chicago, Chicago, IL, United States; Department of Microbiology and Immunology, College of Medicine, University of Illinois Chicago, Chicago, IL, United States

**Keywords:** HSV-1, IL-17, neurotrophic keratitis, optineurin, trigeminal ganglia

## Abstract

Neurotrophic keratitis (NK) arises when trigeminal sensory dysfunction reduces corneal sensation and compromises epithelial maintenance. Herpes simplex virus type 1 (HSV-1) establishes latency in the trigeminal ganglion (TG) and is a common trigger of acquired NK, yet the host programs that determine whether inflamed ganglia recover or degenerate remain poorly defined. Moreover, experimental models that faithfully capture NK-like neuroimmune pathology are limited. Using a murine ocular HSV-1 infection model, we identify optineurin (OPTN) as a key regulator of trigeminal nerve preservation. Optn^−/−^ mice developed severe corneal opacity and rapid, persistent loss of corneal and whisker sensitivity despite comparable corneal viral titers. Droplet-based single-cell RNA sequencing of TGs at 30 days postinfection revealed reduced recovery of peripheral neuronal transcriptomes and coordinated enrichment of chemokine/NF-κB and Th17/IL-17 gene signatures across neurons, endothelial cells, and myeloid/lymphoid populations. Consistent with these transcriptional programs, IL-17 was elevated in Optn^−/−^ TGs at 30 days, whereas the cornea and draining lymph nodes did not exhibit increased IL-17 production early after infection. Neuronal staining demonstrated loss of the synaptic marker SNCG without increased neuronal death, implicating IL-17–associated inflammation in neuronal dysfunction rather than acute ablation. Together, these findings identify OPTN as a neuroimmune checkpoint that restrains chronic IL-17–linked ganglionic inflammation to preserve sensory function and suggest that OPTN deficiency provides a tractable experimental model for studying HSV-associated NK.

## Introduction

Herpes simplex virus type 1 (HSV-1) is a paradigmatic mucosal pathogen that infects epithelial barrier surfaces and establishes lifelong latency within sensory neurons of the trigeminal ganglion (TG).^1–3^ Primary infection typically occurs at mucosal sites such as the oral or ocular epithelium, where the virus replicates within epithelial cells before entering sensory axons and trafficking retrogradely to the TG.^4^^,^^5^ Following latency, periodic viral reactivation can lead to recurrent mucosal disease and sustained host inflammatory responses. HSV-1 infection of mucosal tissues is associated with a broad spectrum of clinical manifestations, including orolabial lesions, keratitis, stromal inflammation, and in severe cases, neurotrophic keratitis (NK) and encephalitis.^6–8^ Among these conditions, ocular HSV-1 infection represents a leading infectious cause of corneal blindness worldwide,^4^^,^^9^^,^^10^ largely due to recurrent inflammatory disease and progressive damage to corneal sensory nerves. Importantly, HSV-1–induced pathology often reflects dysregulated host immune responses rather than direct viral cytopathic effects alone, highlighting the importance of host mechanisms that maintain tissue homeostasis at mucosal surfaces.^1^^,^^11–13^

The ocular surface represents a specialized mucosal barrier in which epithelial, immune, and neuronal networks function in close coordination to maintain tissue integrity and visual function. Disruption of this balance during HSV-1 infection can result in chronic inflammatory disease, including herpes stromal keratitis and NK.^5^^,^^6^^,^^12^^,^^14^ The latter arises when damage to trigeminal sensory pathways reduces corneal sensation and compromises epithelial maintenance, ultimately leading to impaired wound healing, epithelial breakdown, and progressive corneal opacity.^7^^,^^15^ Despite its clinical significance, the host pathways that determine whether TG recover from infection or progress toward chronic inflammatory neuropathy remain incompletely understood. Progress in this area has been further limited by the lack of suitable experimental models that faithfully recapitulate the pathophysiology of NK.

Among inflammatory pathways implicated in ocular surface pathology, IL-17 stands out for its centrality in mucosal immunology. IL-17 family cytokines are key barrier mediators but can also drive immunopathology when excessive or sustained.^16^^,^^17^ In HSV-1 ocular infection, IL-17 and Th17-associated responses have been shown to contribute to corneal inflammatory disease and opacification, and IL-17 can disrupt corneal epithelial barrier function in other ocular surface inflammatory contexts.^16–22^ However, the upstream host determinants that restrain IL-17–linked chronic inflammation in sensory ganglia, and thereby protect long-term neural function after mucosal infection, remain poorly resolved.

Optineurin (OPTN) is a multifunctional adaptor implicated in selective autophagy, innate signaling, and cell death control.^23–32^ Our prior work has shown that OPTN can act as a host-intrinsic restriction factor during neuroinvasive HSV-1 infection by targeting viral proteins for autophagic degradation and promoting neuronal survival.^24^ Here we test the hypothesis that OPTN is not only antiviral but also tissue-protective in the peripheral nervous system: We show that loss of OPTN precipitates NK-like disease after ocular HSV-1 infection through a chronic IL-17–associated neuroinflammatory program in the TG that is uncoupled from corneal viral burden and associated with sustained sensory dysfunction.

## Materials and methods

### Mice

The C57BL/6J Optn^*−/−*^ mouse model used in this study is outlined elsewhere.^5^^,^^11^ Wild-type C57BL/6J mice were purchased from The Jackson Laboratory (Bar Harbor, ME, USA). Male mice were used in this study and all mice were 8 to 12 weeks old at the beginning of each experiment.

### Viruses

HSV-1 (McKrae strain) used throughout this study was provided by Dr. Homayon Ghiasi (Cedars Sinai Health System, Los Angeles, CA, USA).

### Antibodies and stains

The following antibodies and stains were used in this study: anti-gamma synuclein/SNCG antibody (Abcam, ab55424), In Situ Cell Death Detection Kit, TMR red (Roche), DAPI (Sigma), goat anti-mouse IgG (H + L) cross-adsorbed secondary antibody, Alexa Fluor 488 (Thermo Fisher Scientific), anti-rabbit Alexa Fluor 647 (Thermo Fisher Scientific), and anti-rabbit CD3 antibody [SP7] (BioLegend),

### Infections

The corneal scarification method was used for infection of mice as published previously.^24^^,^^31^^,^^32^ In brief, mice were anesthetized using ketamine (100 mg/kg) and xylazine (5 mg/kg). Proparacaine hydrochloride (1% ophthalmic topical solution) was applied to the corneal surface while the mice were unconscious. Corneal epithelial debridement was performed with a 30-gauge needle, then 1 × 10^5^ PFU HSV-1 McKrae strain in a 5-µL volume of PBS was immediately applied to the cornea.

### Drop-seq generation of single-cell libraries

Generation of single-cell libraries was performed exactly as outlined by Macosko et al.^33^ In summary, TGs were dissociated into single-cell suspensions by digestion with the Papain Dissociation System (Worthington Biochemical Corporation, Lakewood, NJ, USA) according to the manufacturer’s protocol. Cells were mixed into droplets with barcoded beads using a microfluidic chip. Droplets were disrupted and cDNA synthesis and PCR were performed to amplify libraries following library preparation using the Illumina Nextera XT kit. Sequencing was performed by the University of Illinois at Chicago DNA services core facility on an Illumina NextSeq.

### Single-cell RNA sequencing analysis

Quality control, alignment to the mouse genome, and generation of the cell expression matrix was performed on Parktek servers using Partek Flow software according to the developer’s manual. The cell expression matrix was then downloaded and read into RStudio as a SeuratObject for analysis with the Seurat R package according to the Satija Laboratory vignettes. Following generation of a SeuratObject for the wild-type and knockout data, the “FindVariableFeatures” function was used to select the top 2,000 variable features. The wild-type and knockout data were then combined by using the “FindIntegrationAnchors” for the first 20 dimensions followed by “IntergrateData” using the integration anchors. We then used the “ScaleData” functions on the newly combined SeuratObject and ran the “RunPCA” function with “features = VariableFeatures.” We decided to use the first 15 principal components and generated clusters using the “FindNeighbors” function with principal components 1 through 15 followed by “FindClusters” with “resolution = 0.15.” For each cluster, “FindConservedMarkers” was used to identify the biomarkers for each cluster. These biomarkers were used to perform literature-based annotation for cluster cell types. We identified contaminating cell types from the pituitary gland, and these were removed from analysis. To obtain differential gene expression (DGE) data, the “FindMarkers” function was used with the negative binomial model. The PathfindR R package was used according to the publisher’s vignette to identify the enriched KEGG pathways for each cluster using the output of the DGE analyses from Seurat.

### Histology

Method for sample preparation for histological analysis is outlined elsewhere.^34^ Following euthanasia and dissection of mice, tissue was fixed overnight in 4% paraformaldehyde at 4 °C. After fixation, tissue was cryoprotected in a 30% sucrose in PBS solution at 4 °C until the tissue sank to the bottom of the tube. Tissue was then embedded in OCT and frozen on crushed dry ice; 10-µm sections were cut and mounted onto Superfrost Plus microscope slides (Fisher Scientific) using a NX10 Cryostat microtome. Hematoxylin and eosin staining was performed as described elsewhere.^34^ For myelin staining, slides were permeabilized with PBT (PBS with 0.1% Triton X-100) with 10% BSA for 1 hour at room temperature (RT). Slides were washed 3 times with PBT before staining with FluoroMyelin Green Fluorescent Myelin Stain (Invitrogen) at 1:300 dilution and DAPI for 2 hours at RT in darkness. Slides were washed 3 times in PBT before mounting with Vectashield mounting solution (Vector Laboratories) and cover glass.

### Immunofluorescence staining

For immunofluorescence staining, the same preparation as above was followed through the permeabilization and blocking steps. The primary antibody and TUNEL reaction mixture were added to slides and incubated at 37 °C for 1 hour. Slides were washed 3 times in PBS, then the secondary antibody and DAPI were applied in PBS for 1 hour at RT. Slides were washed 3 times in PBS before mounting with Vectashield mounting solution (Vector Laboratories) and cover glass.

For TG tissue staining, FFPE tissue sections were used for immunofluorescence experiments. For dewaxing, slides were heated for 15 to 20 minutes and later placed in xylenol for 20 minutes, then 100% ethanol for 10 minutes, then 90% ethanol for 10 minutes, and last 70% ethanol for 10 minutes. The slides were then washed in dH_2_O for 5 minutes. For antigen retrieval, slides were placed in antigen retrieval solution (Vector Laboratories), and the container was microwaved at high for 5 minutes. For permeabilization, slides were washed with PBS/Triton (0.25%, v/v; PBST) solution twice for 20 minutes. Slides were then blocked in 5% BSA in PBS for 60 minutes at RT. Slides were then stained with primary antibodies such as MAP2 (Cat No. 17490-1-AP, 1:500), CD3 (ab237721, 1:500), and IL-17 (740021M, 1:200) in cell staining buffer and kept in the humid box overnight at 4 °C, followed by 3 washes in TBST. For immunofluorescence, slides were stained with the secondary antibody (1:500 in cell staining buffer) 2 hours at RT followed by staining with DAPI for 15 minutes at RT. Slides were washed 3 more times in TBST, then mounted with ProLong Diamond Antifade Mountant (Invitrogen), sealed with clear nail polish, and visualized by fluorescence microscopy.

### Plaque assay

Titration of HSV-1 McKrae strain was performed by plaque assay as outlined elsewhere.^24^ For ocular washes, samples were collected by pipetting a 10-µL droplet of PBS onto the corneal surface, then drawing the liquid back up into the tip and dispelling the sample into 300 µL of Opti-MEM. The sample was used for the same plaque assay protocol referenced.

### Detection of IL-17^+^ cells in corneas and lymph nodes

Whole eyes and lymph nodes were harvested from mice. The right eye was infected, and the contralateral left eye served as the uninfected control. Corneas were dissected separately from right and left eyes, and 3 to 4 corneas were pooled per sample. Corneas were digested in collagenase D (2 mg/mL; MilliporeSigma, C0130) for 1 hour at 37 °C and mechanically dissociated by trituration to generate single-cell suspensions. Lymph nodes were mechanically dissociated using a 5-mL syringe plunger in a 6-well plate. Cell suspensions were filtered through a 70-μm cell strainer, resuspended in FACS buffer (PBS containing 5% FBS), and counted using a hemocytometer. A total of 1 × 10^6^ cells per sample were plated in U-bottom 96-well plates and stimulated with Cell Activation Cocktail (BioLegend, 423303) for 4 hours at 37 °C according to the manufacturer’s instructions. Fc receptors were blocked with TruStain FcX (BioLegend, 101319), followed by surface staining where applicable. Cells were then fixed and permeabilized using the fixation/permeabilization buffer (Tonbo Biosciences, San Diego, CA [TNB-1020-L050]) according to the manufacturer’s protocol, and stained intracellularly with a fluorophore-conjugated anti–IL-17 antibody (BioLegend, 506937) (1:250 dilution). All antibodies were purchased from BioLegend. Cells were washed and analyzed on an Accuri C6 Plus flow cytometer (BD Biosciences), and data were analyzed using FlowJo software (version 10.0.7; Tree Star).

### Corneal sensitivity assessment

Corneal sensitivity in mice was assessed using a manual esthesiometer (12/100 mm, LUNEAU SAS, France). Measurements were performed in awake animals by evaluating the blink reflex in response to corneal stimulation. The nylon filament was initially extended to its maximum length (6 cm), and the tip was gently applied to the central cornea. In normal conditions, this stimulus elicits a blink response, indicating intact sensitivity. If no blink occurred, the filament length was shortened in 0.5-cm increments, and the test was repeated until a response was observed. Sensitivity was scored based on the filament length required to trigger blinking: values between 5.5 and 6 were considered normal, 5.5 to 3.5 indicated a moderate reduction in sensitivity, and values below 3.5 reflected severe loss of corneal sensitivity.^35^

### Corneal opacity scoring

Corneal opacity was evaluated by a masked observer using a standardized grading scale. A score of 0 indicated a fully transparent cornea with clear visualization of the iris and lens. A score of 1 corresponded to mild haze with both structures still clearly visible. A score of 2 reflected moderate opacity, with the iris and lens discernible but less distinct. A score of 3 indicated marked opacity, where these structures were only faintly visible. A score of 4 represented complete opacity with no visible iris or lens.

### Whisker sensitivity assessment

Whisker sensitivity was evaluated in mice following HSV infection using a manual esthesiometer. Animals were tested while awake. The esthesiometer rod was gently touched to the whiskers 3 times per assessment, and responses were recorded. Sensitivity was categorized as bilateral (normal responses on both sides of the muzzle), unilateral (loss of response on one side), or absent (no response on either side). A minimum of 3 mice per group (*n* ≥ 3) were included in each analysis.

### Software

The software used in this study includes Partek Flow (Partek, Inc), GraphPad Prism 9 (GraphPad Software, San Diego, CA, USA), ZEN 3.1 Blue edition (Carl Zeiss, Jena, Germany), RStudio (RStudio, PBC) with R 4.1.1, and ImageJ.

### Statistical analysis

All statistical analysis and graph making was carried out in GraphPad Prism software, except for flow cytometry histograms and dot plots produced in FlowJo software. Error bars represent ± SEM of at least 3 independent measurements (*n* = 3). Asterisks denote a significant difference, as determined by 2-tailed unpaired Student t-test, Mann–Whitney U test, or log-rank test (*P < 0.05, **P < 0.01, ***P < 0.001) or - one-way ANOVA (****P < 0.0001).

## Results

### OPTN deficiency promotes NK-like disease after ocular HSV-1 infection

**Figure 1.**
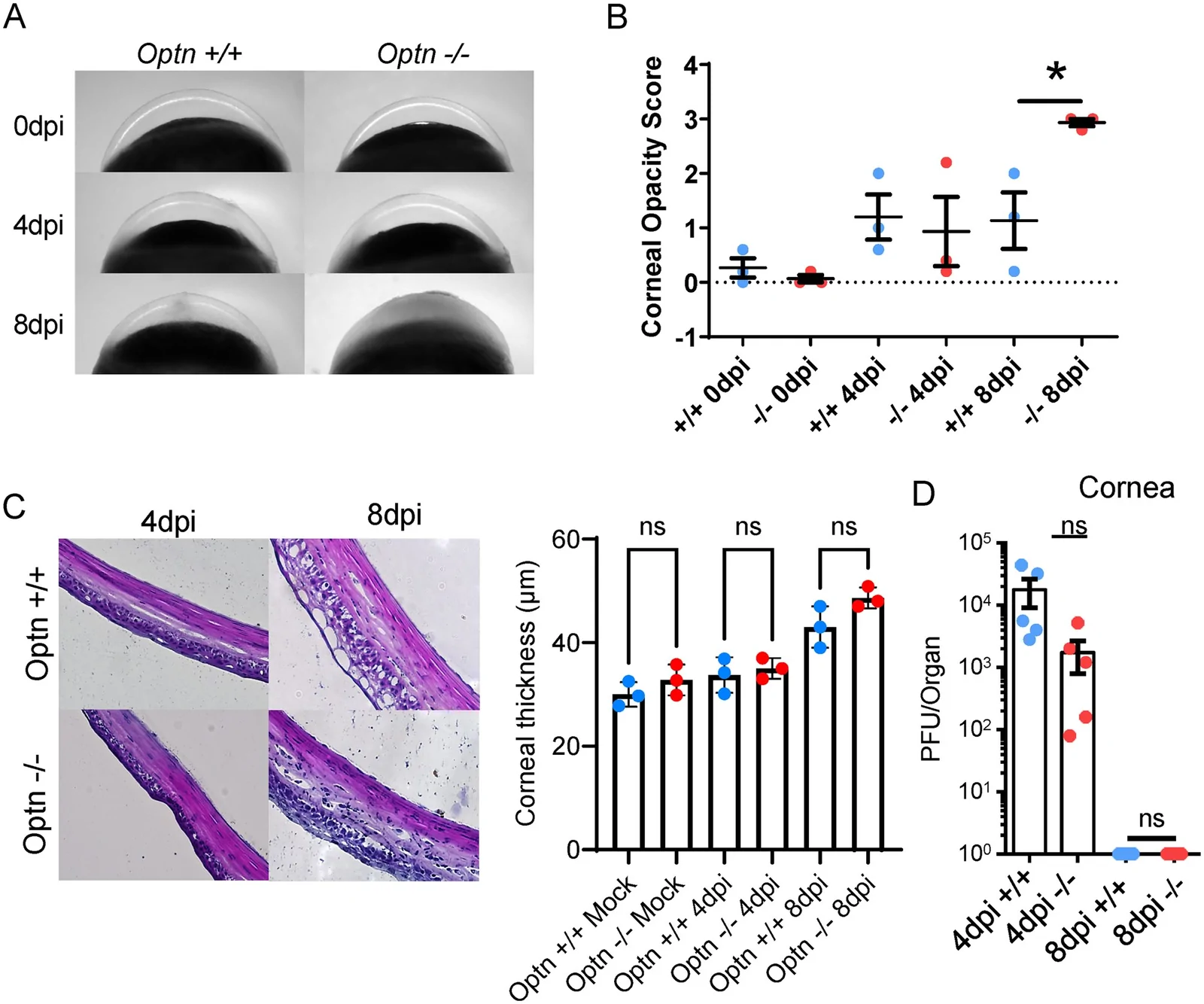
OPTN restricts the severity of neurotropic keratitis. (A) Representative photographs of mouse corneas, highlight the opacification with infection. Scale bar is 1 mm. (B) Quantification of mouse corneal opacity scores following infection. *n* = 3 mice per group. (C) Representative hematoxylin and eosin staining of HSV-1–infected corneas reveal similar levels of inflammation (*n* = 3) and quantification of corneal thickness. (D) Wild-type or Optn knockout mice were infected with 1 × 10^5^ PFU HSV-1, and corneal tissue HSV-1 titers were measured at 4 and 8 days postinfection (dpi) (*n* = 5). For all experiments showing significance, statistical analysis was performed using an unpaired 2-tailed t-test or one-way ANOVA, with significance defined as *P ≤ 0.05; ns, not significant (*P *> 0.05).

Our previous work demonstrated that OPTN protects neurons from HSV-1–induced damage in the TG. Based on these neuroprotective properties, we hypothesized that loss of OPTN would compromise trigeminal sensory integrity and predispose infected animals to NK, a disease characterized by impaired corneal sensation and progressive epithelial pathology. To test this hypothesis, we examined whether ocular HSV-1 infection in Optn^−/−^ mice produces clinical and functional features consistent with NK. Optn^−/−^ and Optn^+/+^ mice were unilaterally infected with HSV-1 by corneal scarification followed by topical viral inoculation. Following infection, Optn^−/−^ animals developed marked corneal opacification that progressed rapidly, resulting in near-complete opacity by 8 days postinfection (dpi) (Fig. 1A, B). Because corneal opacity in herpetic disease can arise from stromal inflammation or scarring, we next assessed corneal tissue architecture and thickness by histological analysis at 4 and 8 dpi. No significant differences in corneal thickness or overall inflammatory pathology were observed between genotypes (Fig. 1C), suggesting that the severe clinical appearance in Optn^−/−^ mice was not explained by exaggerated stromal inflammation.

We next examined whether the pronounced corneal disease in Optn^−/−^ animals reflected increased viral replication in the infected tissue. Viral titers measured from corneal homogenates at both 4 and 8 dpi revealed no significant differences between Optn^−/−^ and control mice (Fig. 1D). Thus, despite the dramatic increase in corneal opacity, OPTN deficiency did not alter corneal viral burden or early inflammatory pathology. These findings indicate that the heightened disease severity observed in Optn^−/−^ animals is unlikely to result from uncontrolled viral replication and instead points toward host determinants affecting neuronal integrity and neuroimmune regulation.

**Figure 2.**
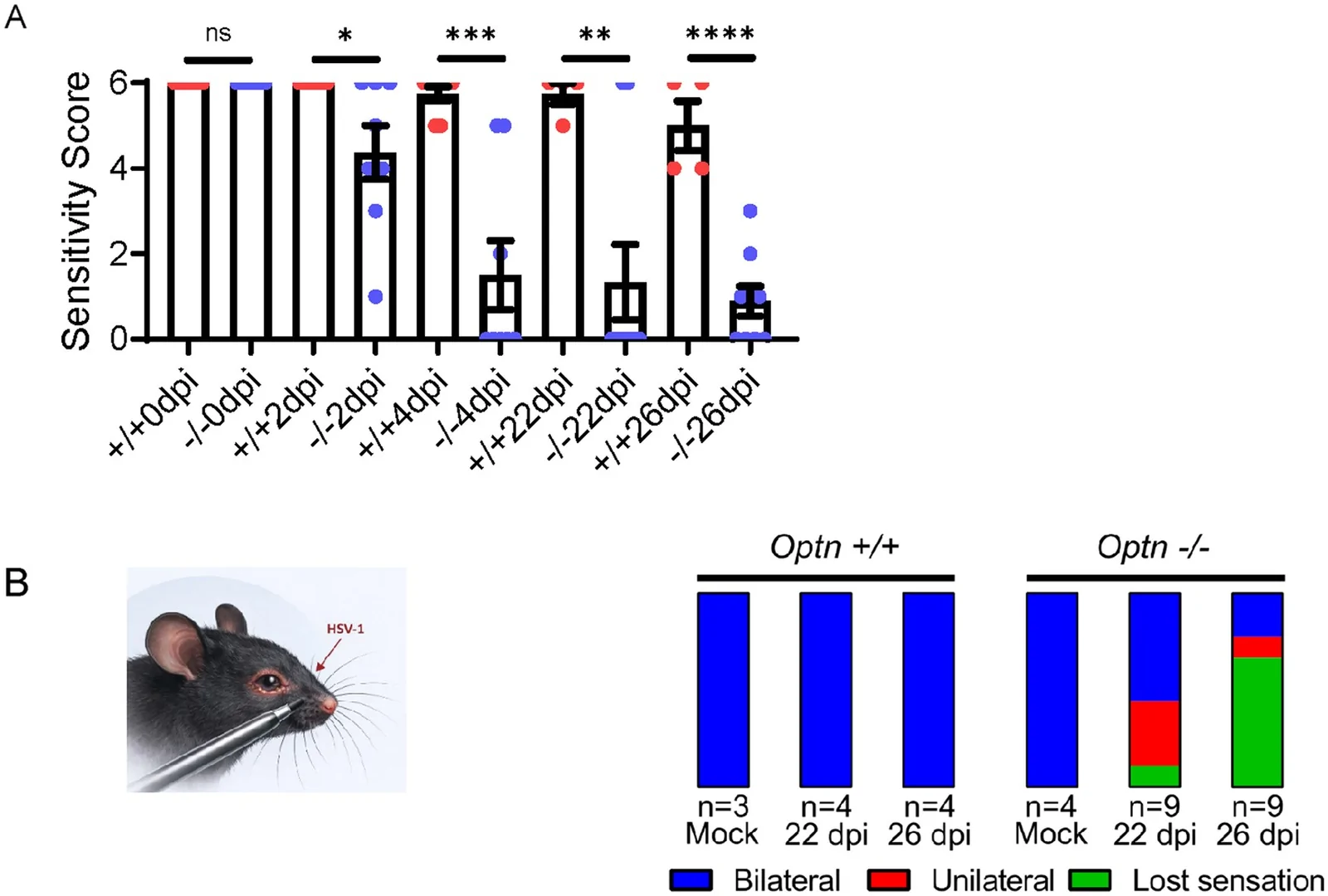
Loss in corneal and facial sensation indicates involvement of trigeminal ganglion. Wild-type or Optn knockout mice were infected with 1 × 10^5^ PFU HSV-1, then (A) schematic representation of cornea corneal sensitivity was measured by blink response at 0, 2, 4, 22, and 26 dpi and (B) whisker sensitivity was reported as bilateral (full sensitivity), unilateral (sensitivity lost on one side of muzzle), or lost sensation. *n* ≥ 3 mice per group. For all experiments showing significance, statistical analysis was performed using an unpaired 2-tailed t-test or one-way ANOVA, with significance defined as *P ≤ 0.05, **P ≤ 0.01, ***P ≤ 0.001, ****P ≤ 0.0001; ns, not significant (*P *> 0.05).

A defining clinical feature of NK is the loss of trigeminal sensory function. We therefore evaluated corneal sensitivity longitudinally following infection. Optn^−/−^ mice exhibited a rapid and sustained loss of corneal sensation, which became evident as early as 4 dpi and persisted through the latest time point examined (26 dpi), whereas Optn^+/+^ animals retained substantially greater corneal sensitivity (Fig. 2A). To determine whether this sensory impairment extended beyond the infected cornea, we also assessed whisker sensitivity as an additional trigeminal sensory readout. Strikingly, Optn^−/−^ mice displayed bilateral whisker sensory deficits by 26 dpi (Fig. 2B). The combination of severe corneal opacity in the absence of increased viral replication, coupled with rapid and persistent loss of corneal and whisker sensation, strongly supports the development of an NK-like phenotype in Optn^−/−^ animals following ocular HSV-1 infection. This finding also suggests that HSV infection may induce long-term deleterious effects on sensory neurons or nerves, leading to persistent whisker sensitivity loss, potentially due to incomplete recovery from damage sustained during the active phase of infection. Overall, these results suggest that OPTN is required to preserve trigeminal sensory integrity during HSV-1 infection and that its loss predisposes to sensory neuropathy-driven corneal disease.

### Single-cell transcriptomic profiling reveals persistent neuroimmune remodeling in OPTN-deficient TG during chronic disease

The combination of increased ocular pathology in Optn^−/−^ mice, unchanged corneal viral titers, and persistent loss of corneal and whisker sensitivity suggested that OPTN deficiency drives long-lasting pathological remodeling within the TG that compromises peripheral sensory neuron function. We therefore hypothesized that loss of OPTN promotes a chronic neuroimmune state within the TG that disrupts neuronal homeostasis and compromises peripheral sensory function following ocular HSV-1 infection.

**Figure 3.**
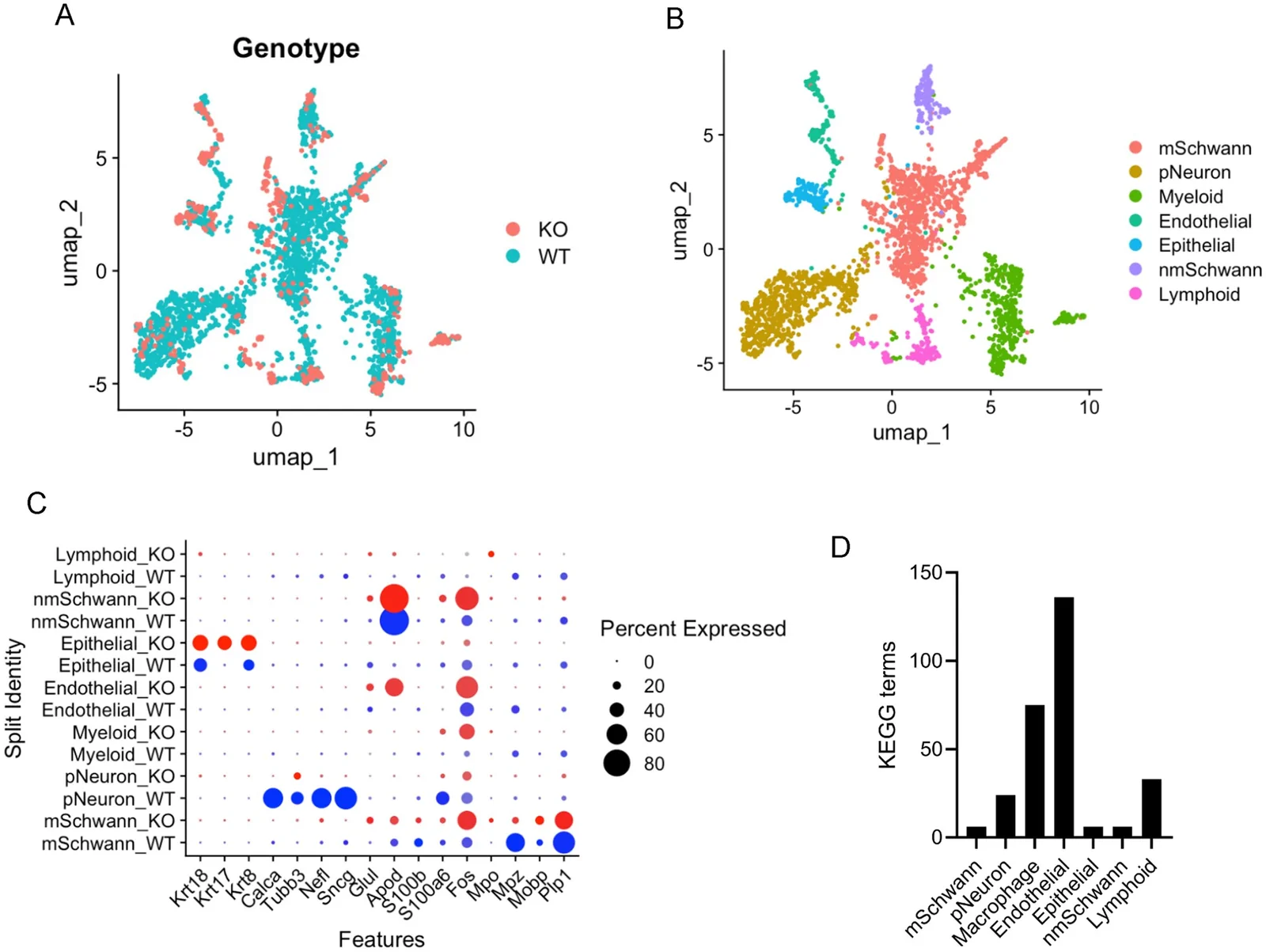
scRNA-seq of 30 dpi trigeminal ganglion (TG) reveal differences between WT and KO mice. scRNA-seq was performed on 3 pooled mouse TG at 30 dpi. (A) UMAP analysis reveals the distribution of cells from Optn^+/+^ (WT) and Optn^−/−^ (KO) across clusters in the dataset. (B) UMAP analyses annotated with putative cell types based on highly represented cluster biomarkers. (C) Dot plot highlighting the expression of several cell type markers by cell type and genotype. (D) Graph of the number of KEGG pathways detected in an enrichment analysis using the differentially expressed genes between KO and WT cells within each cluster.

To investigate this possibility, we performed droplet-based single-cell RNA sequencing (Drop-seq) on TG tissue collected at 30 dpi. This time point was selected to capture transcriptional programs associated with the chronic phase of disease, after resolution of the acute corneal infection. TG from Optn^+/+^ and Optn^−/−^ mice were dissociated to generate single-cell suspensions, and 3 TGs were pooled per genotype to construct cDNA libraries. This approach allowed us to compare transcriptional landscapes between animals that largely recovered from infection (Optn^+/+^) and those exhibiting persistent sensory dysfunction and ocular pathology (Optn^−/−^). In total, 2,746 cells were sequenced and annotated using established literature-based markers. Major TG-resident cell populations were readily identified, including peripheral neurons (pNeurons), myelinating Schwann cells (mSchwann), nonmyelinating Schwann cells (nmSchwann), macrophages, endothelial cells, epithelial cells, and lymphoid cells (Fig. 3A–C). Dimensionality reduction using uniform manifold approximation and projection (UMAP) revealed a notable reduction in the proportion of peripheral neuronal transcriptomes recovered from Optn^−/−^ TG compared with Optn^+/+^ controls. This reduction is consistent with the sensory deficits observed in vivo and suggests impaired neuronal transcriptional recovery or maintenance in the absence of OPTN.

To further understand the molecular basis of these changes, we performed DGE and pathway enrichment analyses within each major cell population postinfection. These analyses revealed widespread transcriptional remodeling across multiple TG compartments, with the most prominent pathway enrichments observed in peripheral neurons, macrophages, endothelial cells, and lymphoid cells in Optn^+/+^ and Optn^−/−^ TG postinfection (Fig. 3D). Notably, many of the differentially expressed pathways were associated with inflammatory signaling, immune activation, and leukocyte recruitment, indicating the emergence of a persistent neuroimmune environment within the TG of animals. Evidently, the loss of OPTN is associated with reduced neuronal transcriptome recovery and coordinated inflammatory transcriptional programs across neuronal and nonneuronal cell populations within the TG. These changes provide a potential mechanistic explanation for the sustained sensory dysfunction observed following ocular HSV-1 infection and support a model in which OPTN restrains chronic neuroimmune remodeling to preserve trigeminal neuronal integrity.

### Pathways linked to leukocyte recruitment and IL-17 signaling regulate inflammatory niche within the TG

**Figure 4.**
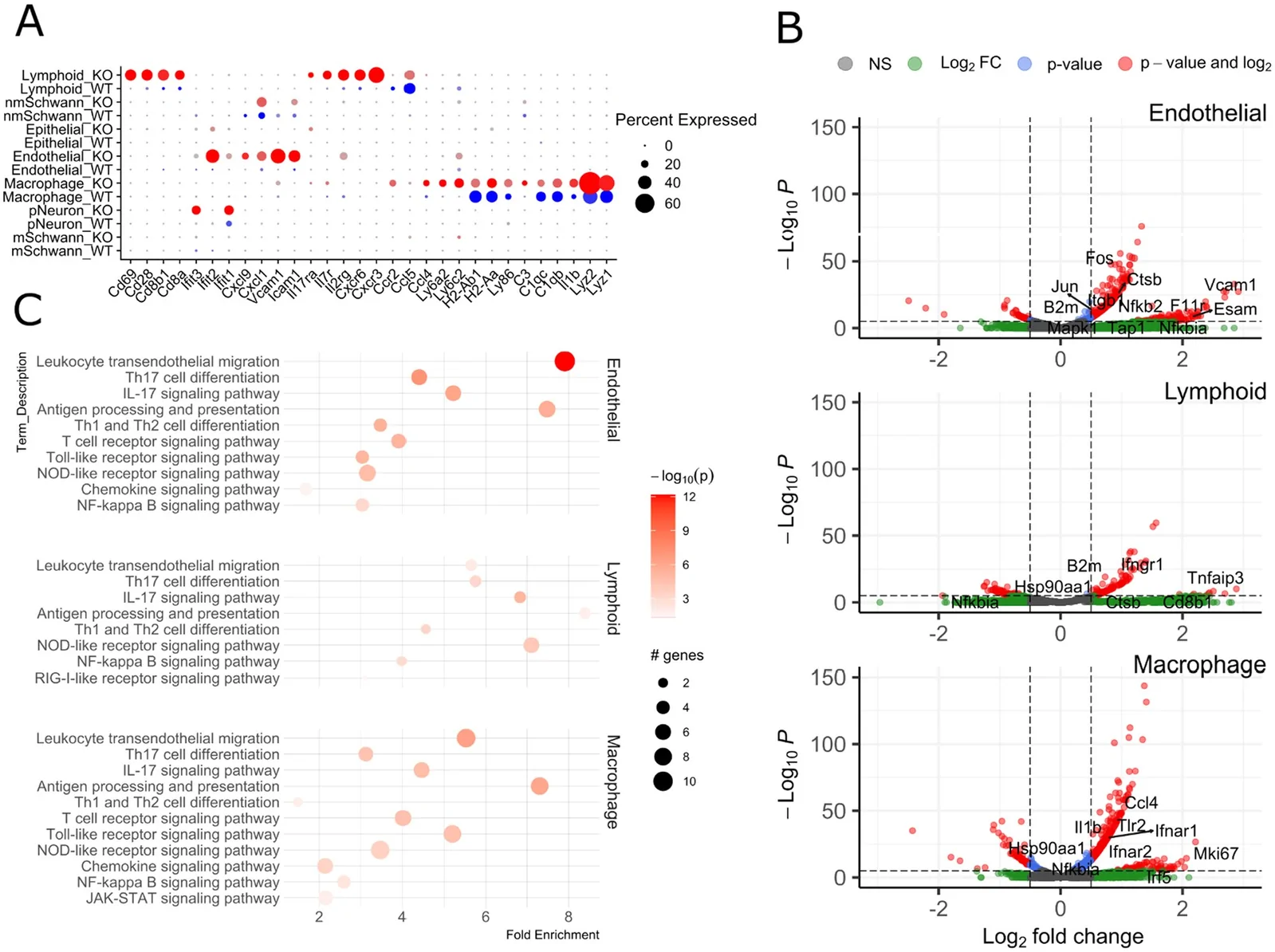
Single-cell analysis reveals differences in immune expression signatures and differentially regulated pathways. (A) Dot plot highlighting the expression of several immune system–related markers by cell type and genotype. (B) Volcano plots of differentially expressed genes in lymphoid, macrophage, or endothelial cell types. (C) Dot plot of significantly enriched immune-related pathways detected in lymphoid, macrophage, or endothelial cell types. FC, fold change; NS, non-significant.

To further define the inflammatory programs associated with OPTN deficiency, we examined immune-related transcriptional signatures across major TG cell populations. Several immune markers were enriched in cell types derived from Optn^−/−^ TG, consistent with the chronic inflammatory phenotype observed in vivo, including persistent keratitis and sensory dysfunction (Fig. 4A). Macrophages from both Optn^+/+^ and Optn^−/−^ TG expressed canonical innate immune and antigen presentation genes, including Lyz1/2, C1qb, C1qc, C3, Ly86, and MHC class II genes (H2-Aa, H2-Ab1). However, macrophages from Optn^−/−^ TG showed increased expression of inflammatory and recruitment-associated genes such as Ccr2, Ccl4, Ly6a, and Ly6c2, suggesting a more activated and inflammatory macrophage phenotype. Endothelial cells from Optn^−/−^ TG exhibited elevated expression of interferon-stimulated genes (Ifit1, Ifit2, Ifit3) as well as chemokines and adhesion molecules involved in leukocyte trafficking, including Cxcl9, Cxcl1, Icam1, and Vcam1. These transcriptional changes are consistent with endothelial activation and suggest the establishment of a permissive gateway for recruitment of circulating immune cells into the TG. Lymphoid populations in Optn^−/−^ TG also displayed transcriptional features consistent with increased immune activation and infiltration. Compared with Optn^+/+^ controls, lymphoid cells from Optn^−/−^ TG showed increased expression of activation markers such as Cd69, Cd28, Cd8a, and Cd8b1, indicating the presence of activated CD8^+^ T cells. In addition, elevated expression of Il17ra, Il7r, Il2rg, Cxcr6, and Cxcr3 further suggested recruitment or expansion of inflammatory T-cell populations within the ganglion.

To determine whether these transcriptional differences reflected broader inflammatory signaling programs, we performed DEG and KEGG pathway enrichment analyses across the major TG cell types. Multiple immune and inflammatory pathways were significantly enriched in Optn^−/−^ TG, including leukocyte transendothelial migration, NF-κB signaling, chemokine signaling, Th17 differentiation, and IL-17 signaling (Fig. 4B, C). Collectively, our results indicate that OPTN deficiency is associated with coordinated inflammatory activation across neuronal and stromal compartments of the trigeminal ganglion. The enrichment of pathways linked to leukocyte recruitment and IL-17 signaling suggests the establishment of a persistent inflammatory niche within the TG that may contribute to neuronal dysfunction and the development of neurotrophic keratitis following ocular HSV-1 infection.

### Increased IL-17 expression in the TG contributes to persistent neuroinflammation

**Figure 5.**
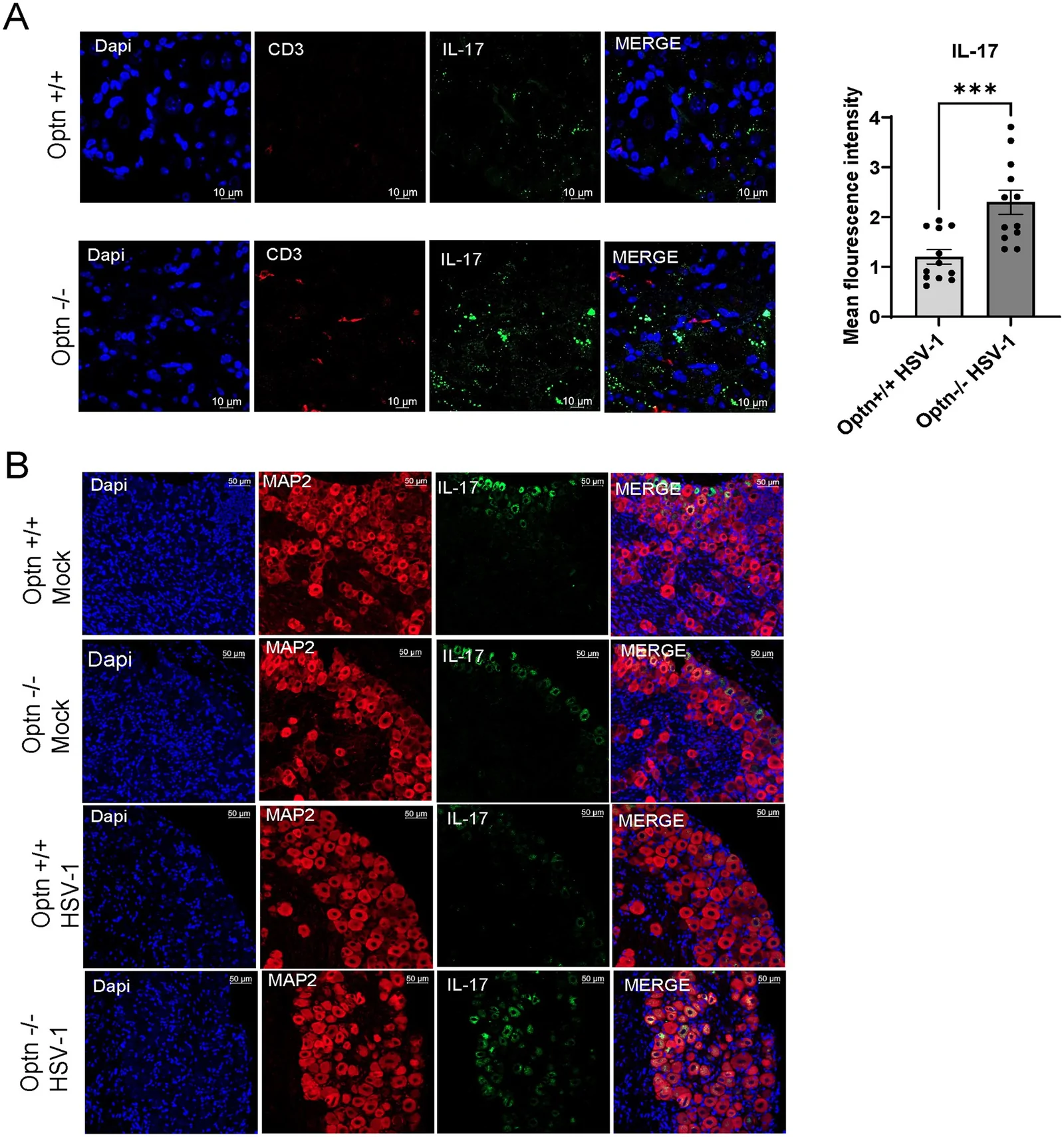
Immunofluorescence confirms IL-17 pathology in trigeminal ganglion (TG). (A) Representative images from Optn^+/+^ and Optn^−/−^ indicating IL-17 (green) and CD3 (red) expression in TG at 30 dpi and quantification of IL-17 expression. (B) Representative images from Optn^+/+^ and Optn^−/−^ mock and HSV-1 indicating IL-17 (green) and MAP2 (red) expression in TG at 30 dpi. For all experiments showing significance, statistical analysis was performed using an unpaired 2-tailed t-test or one-way ANOVA, with significance defined as ***P ≤ 0.001.

To validate the transcriptional signatures identified by single-cell RNA sequencing (scRNA-seq), we next examined IL-17 expression within TG following ocular HSV-1 infection. Immunofluorescence staining revealed a marked increase in IL-17 signal within TG tissue from Optn^−/−^ mice at 30 dpi compared with Optn^+/+^ controls (Fig. 5A, B). Consistent with the immune activation signatures detected in the single-cell dataset, TG sections from Optn^−/−^ animals also displayed an increased presence of T cells. Unexpectedly, however, IL-17 staining showed limited colocalization with T cells within the ganglion. These observations indicate that although T cell infiltration is increased in the Optn^−/−^ TG, conventional T cells may not represent the primary source of IL-17 detected at this chronic stage of infection. Instead, IL-17 may originate from alternative immune populations or from nonclassical lymphoid sources within the inflammatory TG environment. Therefore, based on prior literature we determined whether neurons are the source of IL-17 in TG. We observed MAP2 (neuronal marker) co-localized with IL-17 in TG of both Optn^+/+^ and Optn^−/−^ mock and infected TG at 30 dpi (Fig. 5B). Together, these findings independently validate the scRNA-seq prediction of enhanced IL-17–associated signaling within the trigeminal ganglion and demonstrate that OPTN deficiency is associated with the establishment of an IL-17–enriched inflammatory niche during the chronic phase of ocular HSV-1 infection. This localized cytokine environment may contribute to persistent neuroinflammation and impaired neuronal function that underlies the NK-like phenotype observed in Optn^−/−^ animals.

### Elevated IL-17 signaling in OPTN-deficient mice arises independently of peripheral immune responses

**Figure 6.**
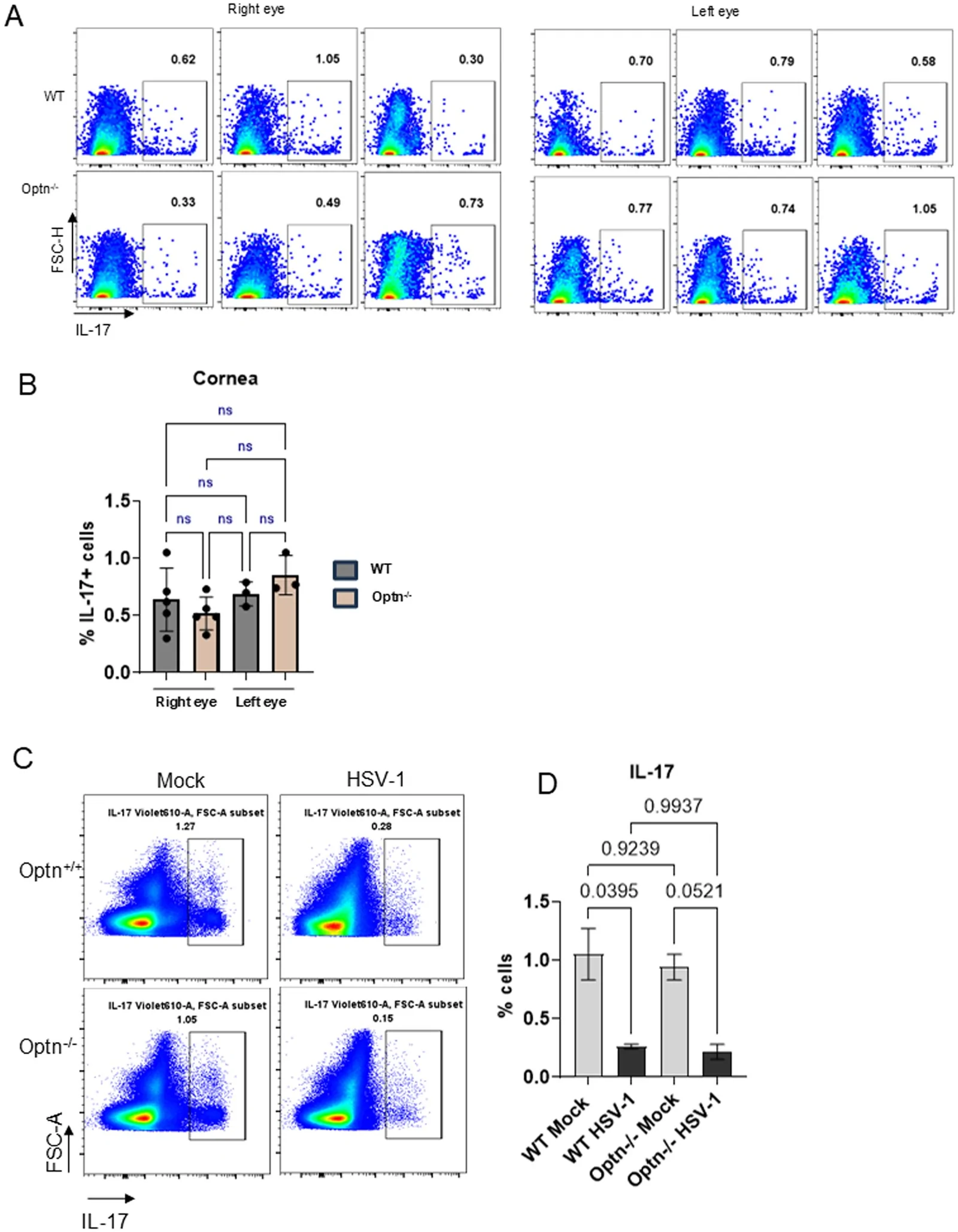
T cell–independent driven IL-17 pathology at ocular surface. (A) Representative FACS plots for corneal cells producing IL-17 from Optn^+/+^ and Optn^−/−^ upon 8 dpi. (B) Statistical analysis for (A) where *n* = 3 to 5. (C) Representative FACS plots for immune cells in cervical lymph nodes producing IL-17 from Optn^+/+^ and Optn^−/−^ upon 8 dpi. (D) Statistical analysis for (C) where *n* = 2. For all experiments showing significance, statistical analysis was performed using an unpaired 2-tailed t-test or one-way ANOVA. ns, not significant (*P *> 0.05).

Given the increased IL-17 signal detected within the TG of Optn^−/−^ mice, we next asked whether this cytokine originated from peripheral immune responses induced by ocular HSV-1 infection. To address this possibility, we quantified IL-17 production by immune cells isolated from the primary site of infection and associated draining lymphoid tissue. At 8 dpi, when peripheral immune activation is expected to be robust, immune cells isolated from the infected cornea were analyzed for IL-17 production. No significant differences in IL-17 expression were observed between Optn^+/+^ and Optn^−/−^ mice (Fig. 6A, B). Similarly, analysis of immune cells isolated from the deep cervical lymph nodes, which drain the ocular surface, revealed comparable IL-17 production between genotypes (Fig. 6C, D). These findings indicate that OPTN deficiency does not amplify IL-17 responses within peripheral immune compartments during the acute phase of infection. Instead, the absence of genotype-dependent differences in IL-17 production at the site of infection and in draining lymph nodes argues against a peripheral immune origin for the elevated IL-17 detected in Optn^−/−^ TG. Collectively, these results support a model in which IL-17 dysregulation arises locally within the TG during the chronic phase of infection. This localized cytokine environment is consistent with the persistent neuroimmune remodeling observed in Optn^−/−^ TG and suggests that OPTN functions to restrain the emergence of an IL-17–associated inflammatory niche that may contribute to neuronal dysfunction and the development of NK following ocular HSV-1 infection.

### OPTN preserves neuronal integrity and prevents neuronal loss in TG

**Figure 7.**
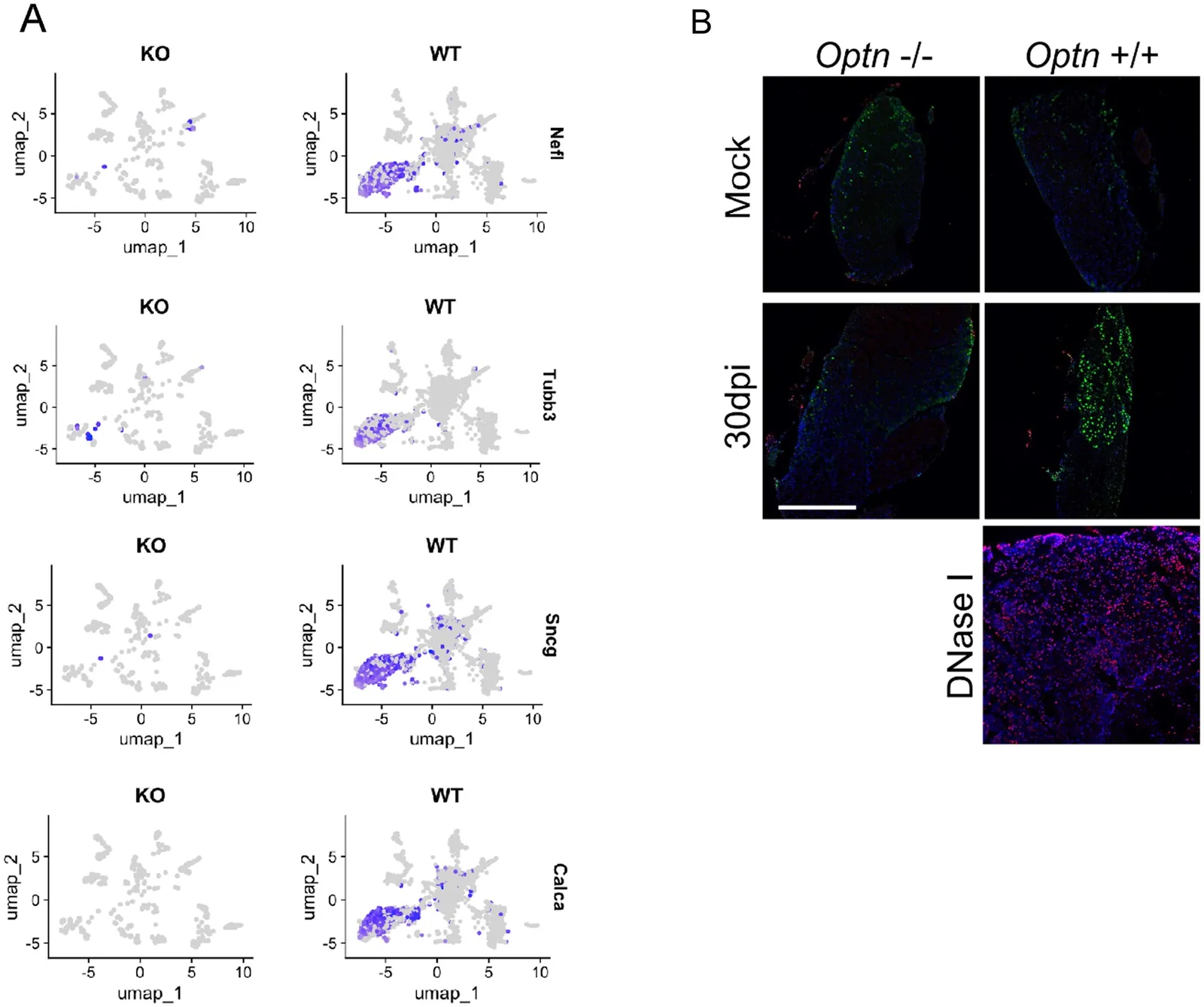
Optn^−/−^ neurons lose neuronal marker expression. (A) UMAP analysis for peripheral neuron markers split by genotype reveals distribution of markers across dataset. (B) Representative staining for SNCG or TUNEL in mock and 30 dpi mouse trigeminal ganglion tissue. Scale bar is 500 µm.

Given the pronounced IL-17–associated inflammatory environment observed in Optn^−/−^ TG, we asked whether this chronic neuroimmune state results in neuronal loss or functional impairment of peripheral sensory neurons. Transcriptomic analysis revealed a marked reduction in peripheral neuronal transcriptomes in Optn^−/−^ TGs, suggesting compromised neuronal activity or maintenance. To determine whether these changes reflected neuronal death, we assessed neuronal integrity in TG sections at 30 dpi. Despite the reduction in neuronal transcriptomes and neuronal marker expression detected by single-cell analysis, we did not observe significant differences in neuronal cell death between Optn^+/+^ and Optn^−/−^ mice (Fig. 7A, B). We next examined expression of the neuronal and synaptic marker SNCG (gamma-synuclein), which is associated with neuronal identity and synaptic function. Immunofluorescence analysis revealed a significant reduction in SNCG staining in Optn^−/−^ TG compared with Optn^+/+^ controls at 30 dpi (Fig. 7A, B). The loss of this synaptic marker suggests disruption of neuronal molecular identity and synaptic integrity rather than widespread neuronal ablation. Considered together, these findings indicate that the IL-17–associated inflammatory environment that emerges in the absence of OPTN primarily affects neuronal function rather than inducing acute neuronal death. This functional impairment is consistent with the persistent sensory deficits observed in Optn^−/−^ animals and supports a model in which chronic neuroimmune remodeling within the TG disrupts neuronal connectivity and sensory signaling, ultimately contributing to the development of neurotrophic keratitis following ocular HSV-1 infection.

## Discussion

In this study, we identify OPTN as a critical host regulator that protects against HSV-1–induced NK by maintaining TG homeostasis and restraining IL-17–associated peripheral nervous system inflammation. Using a murine ocular HSV-1 infection model, we demonstrate that loss of OPTN results in severe corneal opacification and persistent loss of corneal and whisker sensitivity, clinical hallmarks of NK. Notably, these manifestations occurred in the absence of increased corneal viral replication or overt differences in corneal inflammatory pathology. These findings indicate that disease severity in OPTN-deficient animals is driven primarily by host-mediated neuropathology rather than uncontrolled infection, positioning OPTN as an important neuroimmune checkpoint that preserves sensory neuron integrity during mucosal viral infection.

Our findings advance understanding of neuroimmune regulation during mucosal viral infection in 3 important ways. First, we identify OPTN as a previously unrecognized neuroprotective factor required to maintain trigeminal sensory function following HSV-1 infection. Second, we demonstrate that IL-17–associated inflammatory signaling can arise locally within sensory ganglia and contribute to neuronal dysfunction independently of classical peripheral immune responses. Third, we establish an experimental system that recapitulates key features of NK, providing a platform to investigate the mechanisms linking mucosal infection, ganglionic inflammation, and sensory neuropathy.

Damage to trigeminal sensory pathways represents a central event in the development of NK.^36^^,^^37^ Corneal sensory neurons regulate epithelial maintenance, wound healing, and protective reflexes; thus, disruption of trigeminal nerve function can lead to progressive epithelial disease even in the absence of active infection. Consistent with this paradigm, OPTN-deficient mice developed rapid and persistent loss of corneal sensation following HSV-1 infection, accompanied by bilateral whisker sensory deficits, indicating widespread trigeminal sensory dysfunction. These symptoms were still detectable at 26 days postinfection. This is particularly intriguing, especially given that the virus is expected to be in a latent state at this time point. This finding suggests that sensory dysfunction may persist beyond active viral replication and could reflect lasting neuronal alterations or immune-mediated effects within specific TG neuronal populations or along peripheral projections of the trigeminal nerve, particularly the maxillary branch of the trigeminal nerve and ophthalmic branch of the trigeminal nerve that innervate the whisker pad. Importantly, these neurological impairments occurred despite comparable viral titers between genotypes, supporting a model in which dysregulated host responses within the TG, rather than enhanced viral cytopathology at the ocular surface, drive disease progression. Single-cell transcriptomic analysis revealed extensive neuroimmune remodeling within TG lacking OPTN. OPTN-deficient ganglia displayed reduced recovery of peripheral neuronal transcriptomes together with coordinated activation of inflammatory pathways across multiple cell types, including macrophages, endothelial cells, and lymphoid populations. Among the most prominent transcriptional signatures were chemokine signaling, NF-κB signaling, leukocyte trafficking programs, and pathways associated with Th17 differentiation and IL-17 signaling. These findings suggest that OPTN restrains the emergence of a chronic inflammatory niche within the TG during HSV-1 infection. In particular, endothelial cells in OPTN-deficient ganglia exhibited increased expression of adhesion molecules and chemokines associated with leukocyte recruitment, consistent with the establishment of an inflammatory gateway that facilitates immune cell entry into neural tissue.

A central insight emerging from this study is the prominent role of IL-17–associated signaling within the TG during chronic infection. IL-17 family cytokines are key regulators of mucosal immunity and contribute to host defense at epithelial barrier surfaces.^16^^,^^18^^,^^20^^,^^21^^,^^38^ However, excessive or sustained IL-17 signaling can drive tissue pathology and chronic inflammation. While IL-17 has previously been implicated in HSV-1–induced corneal inflammation, our findings reveal that IL-17–associated signaling also operates within sensory ganglia and may contribute to neuronal dysfunction following mucosal viral infection. This observation highlights TG as an important neuroimmune interface in which barrier-derived immune responses intersect with neuronal biology.

Multiple lines of evidence indicate that the elevated IL-17 observed in OPTN-deficient mice arises locally within the TG rather than from peripheral immune compartments. Immunofluorescence analyses demonstrated increased IL-17 expression within ganglionic tissue, yet the cytokine signal showed limited colocalization with infiltrating T cells. Furthermore, flow cytometric analysis revealed comparable IL-17 production in the infected cornea and draining cervical lymph nodes between genotypes. These findings argue against enhanced peripheral Th17 responses as the source of IL-17 dysregulation and instead support a model in which IL-17 signaling emerges locally within the ganglion microenvironment (Fig. 5B). Such cytokine production may originate from noncanonical immune populations or from inflammatory programs operating within resident ganglionic neurons during chronic infection.

Despite the pronounced inflammatory environment present in OPTN-deficient TG, neuronal cell death was not a prominent feature at the chronic stage examined in this study. Instead, neurons exhibited a marked reduction in the synaptic marker SNCG, indicating disruption of neuronal molecular identity and synaptic integrity. These findings suggest that chronic IL-17–associated inflammation primarily impairs neuronal function rather than inducing immediate neuronal ablation. Functional silencing of trigeminal sensory neurons would be expected to disrupt corneal innervation and sensory signaling, thereby providing a mechanistic explanation for the persistent sensory deficits and NK-like pathology observed in OPTN-deficient animals. Future studies will use targeted approaches, such as neuron-specific IL-17 deletion in the context of OPTN deficiency or spatially restricted IL-17 blockade within the TG, to address these questions more directly. Likewise, distinguishing between sensory and sympathetic nerve alterations remains an important question that should be addressed in future studies. Beyond revealing a peripheral neuroprotective role for OPTN, our findings also establish a tractable experimental framework for studying NK. Experimental models that faithfully recapitulate NK-like sensory neuropathy remain limited. The OPTN-deficient HSV-1 infection model described here reproduces several defining features of NK, including persistent corneal hypoesthesia, progressive corneal opacity, and neuronal dysfunction occurring independently of increased viral replication. This system therefore provides a valuable platform for investigating how chronic neuroimmune interactions within sensory ganglia contribute to corneal disease and for evaluating therapeutic strategies aimed at preserving neuronal integrity. Collectively, our findings reveal that OPTN in TG regulates the neuroimmune microenvironment, linking ocular surface mucosal infection to peripheral nervous system dysfunction and corneal pathology.

## Data Availability

The data that support the findings of this study are available from the corresponding author upon request.
